# A Review of Ionizing Radiation-Induced Senescence of Bone Marrow Mesenchymal Stem/Stromal Cells: Mechanisms and Therapeutic Strategies

**DOI:** 10.3390/cimb48020196

**Published:** 2026-02-10

**Authors:** Xiaoliang Li, Maoshan Chen, Yangyang Zhang, Jiuxuan Li, Lixin Xiang, Yanni Xiao, Yang Xiang, Li Chen, Qian Ran, Zhongjun Li

**Affiliations:** 1 Department of Blood Transfusion, Laboratory Medicine Center, The Second Affiliated Hospital, Army Medical University, Chongqing 400037, China; lixiaoliang@tmmu.edu.cn (X.L.); maoshanchen@tmmu.edu.cn (M.C.); zhangyangyang_cn@163.com (Y.Z.); li_jiuxuan@126.com (J.L.); zuling19@tmmu.edu.cn (L.X.); jinshanxilin@163.com (Y.X.); xiangy105@163.com (Y.X.); chenli200401@163.com (L.C.); 2 Hematopoietic Acute Radiation Syndrome Medical and Pharmaceutical Basic Research Innovation Center, Ministry of Education of the People’s Republic of China, Chongqing 400037, China

**Keywords:** bone marrow mesenchymal stem/stromal cell, ionizing radiation, senescence, DNA damage, mitochondrial dysfunction

## Abstract

Bone marrow mesenchymal stem/stromal cells (BM-MSCs) are important components of bone marrow, possessing multipotent differentiation potential and the ability to support hematopoiesis. Exposure to ionizing radiation (IR) induces cellular damage in BM-MSCs, such as DNA lesions and mitochondrial dysfunction. Despite their relative radioresistance, most surviving BM-MSCs enter senescence post-irradiation. This senescent state disrupts the bone marrow niche, impairs stem cell proliferation and differentiation, and contributes to acute radiation syndrome (ARS) and myelosuppression. To clarify the impact of IR on BM-MSCs, this review systematically summarizes the general mechanisms of radiation-induced cellular senescence, examines the effects of different radiation types (e.g., gamma rays, X-rays, and heavy-ion radiation) and doses on BM-MSCs senescence, and outlines senotherapeutic strategies targeting BM-MSCs senescence. The analysis indicates that the senescence of BM-MSCs caused by IR is type- and dose-dependent. The review identifies key factors in IR-induced BM-MSCs senescence to guide targeted interventions, highlighting the need for future studies to elucidate the underlying mechanisms of IR-induced BM-MSCs senescence.

## 1. Introduction

Bone marrow mesenchymal stem/stromal cells (BM-MSCs) are pluripotent stem cells located in the stromal compartment of the bone marrow (BM), a soft and highly vascularized tissue that is responsible for hematopoiesis and immune cell production [1]. Ionizing radiation (IR) from nuclear accidents, radiotherapy, and prolonged spaceflight constitutes a significant public health concern due to its potential to cause detrimental biological effects. BM is highly sensitive to IR, with the resultant damage manifesting chiefly as myelosuppression [2,3]. Nevertheless, BM-MSCs are relatively radiation-resistant compared to other cells in the BM, even at high radiation doses [4]. This resistance may be due to high expression of anti-apoptotic proteins, low expression of pro-apoptotic proteins, high anti-oxidative capacity and effective DNA damage response (DDR) [5,6]. Notably, BM-MSCs that survive irradiation subsequently enter senescence [7]. Once senescent, BM-MSCs undergo irreversible cell cycle arrest. Simultaneously, they exhibit an enlarged and flattened morphology. Furthermore, these cells develop a senescence-associated secretory phenotype (SASP), which is driven by epigenetic reprogramming and persistent DNA-damage signaling [8]. SASP comprises the secretion of pro-inflammatory cytokines, chemokines, and matrix metalloproteinases (MMPs), all of which alter the microenvironment [9]. These molecular changes lead to the down-regulation of niche factors, skewed differentiation toward adipogenesis, and loss of paracrine immunosuppressive activity [10]. Consequently, the hematopoietic-supportive capacity and therapeutic potential of senescent BM-MSCs in regenerative medicine are markedly diminished. Moreover, BM-MSC senescence contributes to the progression of hematological malignancies such as acute myeloid leukemia (AML), chronic lymphocytic leukemia (CLL), and multiple myeloma (MM) by fostering a pro-inflammatory microenvironment and supporting leukemic stem-cell survival [11]. Targeting IR-induced BM-MSC senescence represents a promising therapeutic strategy for mitigating acute radiation syndrome (ARS) and subsequent long-term myelosuppression. However, previous reviews on MSC senescence have primarily focused on general mechanisms or broad pathological contexts [7,12,13], leaving critical gaps in understanding how specific radiation parameters—such as type (e.g., γ-rays, X-rays, and charged particles) [14] and dose (e.g., low-dose adaptive responses vs. high-dose irreversible damage) [15]—modulate BM-MSC senescence pathways. Unlike existing works, this review explicitly compares the effects of various radiation types, based on their linear energy transfer (LET) characteristics and dose response, and summarizes both existing and emerging therapeutic strategies for cellular senescence. It aims to provide new insights for precisely targeting radiation-induced senescence in BM-MSCs. Here, we first summarize the general mechanisms of IR-induced cellular senescence, then focus on how specific radiation types and doses regulate BM-MSC senescence to elucidate the underlying molecular mechanisms. Finally, we discuss therapeutic strategies for cellular senescence, offering a refined perspective for developing targeted interventions.

## 2. IR-Induced Cellular Senescence

IR is a powerful form of energy, including gamma-rays (γ-rays), X-rays, neutrons and heavy-ion particles, and has been widely used in various industrial and medical fields [16]. It inflicts multifaceted cellular damage through both direct energy deposition and indirect radical-mediated mechanisms. The ultimate cell fate (survival, senescence, or death) is determined by the severity of the damage and the efficiency of the cellular repair response [17,18]. This damage exhibits pronounced subcellular specificity. For example, in the nucleus, direct attacks and reactive oxygen/nitrogen species (ROS/RNS) lead to various types of DNA damage, causing genomic instability [19]; in mitochondria, mtDNA damage and lipid peroxidation promote permeability-transition-pore opening, collapsing the membrane potential and amplifying oxidative stress [20]; in the endoplasmic reticulum (ER), protein oxidation and calcium dyshomeostasis induce ER stress and an unfolded protein response [21]; and in the plasma membrane and lysosomes, lipid peroxidation compromises integrity, increasing lysosomal permeability and causing enzyme leakage [22]. The accumulated, unrepaired, and incorrectly repaired damage ultimately leads to cellular senescence and death, thereby contributing to acute radiation syndrome (ARS), carcinogenesis, and other health problems [23,24]. Notably, the biological outcome of IR may depend on the dose and type of radiation [25].

Radiation doses and types play a critical role in clinical and aerospace applications. In clinical oncology, for example, heavy ions improve dose distribution in tumor radiotherapy due to the Bragg peak effect, but they also pose challenges such as radiation-induced tissue injury in healthy tissues [26]. Meanwhile, high-dose fractionation approaches like stereotactic body radiotherapy have spurred debate on immune modulation [27], and hybrid strategies (e.g., combining carbon ions with photons) show potential for synergistic cytotoxicity [28]. In aerospace, the mixed radiation environment in space—containing high-energy particles—threatens astronauts’ cardiovascular health in a dose-dependent manner, driving the need for protective measures against high-LET radiation [29].

LET describes the rate at which the energy (in kiloelectronvolt) is transferred per unit length (in micrometer) of the track (keV/μm) [30]. Owing to the high penetration capacity and oxygen-dependent effects, damage caused by low-LET photon radiation (γ-rays and X-rays) is different from that caused by charged particle radiation. It is estimated that approximately 70% of the biological damage produced by photon radiation is attributable to ROS/RNS generated during the radiolysis of water [31]. In contrast, heavy-ion radiation is classified as high-LET radiation and imparts a significantly higher energy density to the molecules along its trajectory [32]. Although its yield of ROS is lower than that of photons, it exhibits higher relative biological effectiveness. This is primarily due to its ability to cause complex and clustered DNA lesions that challenge the cellular repair mechanisms [33]. Consequently, various types of cellular damage induced by IR ultimately determine the cells’ fate, including senescence, apoptosis, necrosis, and other types of cell death [34]. The overall outcome of radiation damage to a cell is governed by key factors, such as absorbed dose, LET, and cell type.

**Figure 1:**
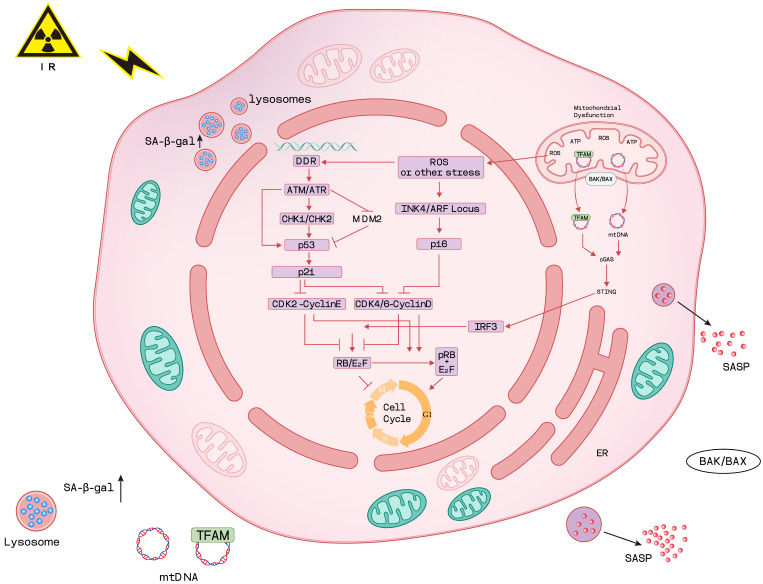
The mechanisms of IR-induced cellular senescence (created with Adobe Illustrator 28.5).

Radiation-induced cellular damage is multifaceted, arising primarily through two core mechanisms—direct ionization of DNA and indirect injury mediated by ROS [35]. These insults initiate a cascade of pathological events, including cell cycle arrest, genome instability, mitochondrial dysfunction, and epigenetic alterations, all of which are associated with cellular senescence [36]. The mechanisms of cellular senescence induced by DNA damage and oxidative lesions will be discussed in the following sections to illustrate how IR triggers these key cellular responses (Figure 1).

### 2.1. DNA Damage-Induced Cellular Senescence

The senescence induced by IR is predominantly driven by the persistence of specific types of DNA damage and the ensuing sustained activation of the DDR. It is well known that IR induces various types of damage, including base and sugar lesions, single-strand breaks (SSBs), and double-strand breaks (DSBs) [33]. Notably, these lesions differ markedly in their capacity to trigger cellular senescence, largely because of variations in their complexity and repair kinetics [37]. Most base and sugar lesions and isolated SSBs can be rapidly removed by base-excision repair (BER) or SSB repair (SSBR). However, when these pathways are saturated or defective, the lesions can further persist and activate the p38MAPK-p16-Rb axis that drives cells toward senescence [38]. Furthermore, persistent SSBs can cause replication fork collapse, giving rise to one-end DSBs and genetic instability [39]. Compared with SSBs, DSBs represent the most detrimental type of DNA lesion due to their harmful impact on genomic integrity and chromosomal stability [40]. Simple DSBs can be repaired primarily through non-homologous end joining (NHEJ) or high-fidelity homologous recombination (HR) [41]. However, complex DSBs (also termed clustered DNA damage), which comprise two or more oxidative base lesions, basic sites, and SSBs clustered around a DSB, are repaired more slowly and with lower fidelity [42]. This inefficient repair results in genomic instability, chromosomal aberrations, and ultimately cell death or senescence [37]. Moreover, recent evidence suggests that radiation-induced damage, particularly in the paternal germline, can be inherited across generations through error-prone DNA repair mechanisms such as polymerase theta-mediated end joining (TMEJ), further amplifying genomic instability in offspring [43]. Additionally, radiation alters the chromatin structure by upregulating histone H1 and heterochromatin proteins, which restrict access to accurate DNA repair machinery like HR, thereby exacerbating senescence and the transgenerational detrimental effects [43].

In response to DNA damage, the phosphatidylinositol-3 kinase-related kinases (PIKKs), including ataxia-telangiectasia and RAD3-related kinase (ATR), ataxia-telangiectasia mutated (ATM) and DNA-dependent protein kinase (DNA-PK), are activated in the DDR pathways [44,45]. ATR is responsible for responding to SSBs [46], while ATM and DNA-PK are usually involved in responding to DSBs [47]. When these kinases are recruited to DNA damage sites, they are activated through phosphorylation to initiate DDR and lead to phosphorylation and activation of numerous downstream targets [48]. For example, checkpoint kinases 1 and 2 (CHK1 and CHK2) are well known downstream effectors of ATR and ATM [49]. CHK1 is mainly activated by ATR, and phosphorylate cell division cycle 25 homolog A (CDC25A) to inhibit cyclin-dependent kinase (CDK) activity. As a result, the activation of CHK1 triggers the intra-S phase and G2/M phase checkpoints [50]. On the other hand, activation of CHK2 by ATM can phosphorylate tumor suppressor protein (TP53, p53) and CDC25A to arrest cell cycle in G1, S or G2/M phase [51]. In addition, phosphorylated forms of the histone variant H2AX (γH2AX) and adaptor protein p53-binding protein 1 (53BP1) are also important substrates of PIKKs [45]. The γH2AX and 53BP1 foci are considered to be reliable markers for DSBs and play a crucial role in the HR and NHEJ repair pathways [45]. Therefore, IR-induced senescence is initiated by persistent DNA damage, with complex DSBs being particularly potent due to challenging repair. Defective resolution of these lesions triggers downstream signaling that promotes cellular senescence.

**Figure 2:**
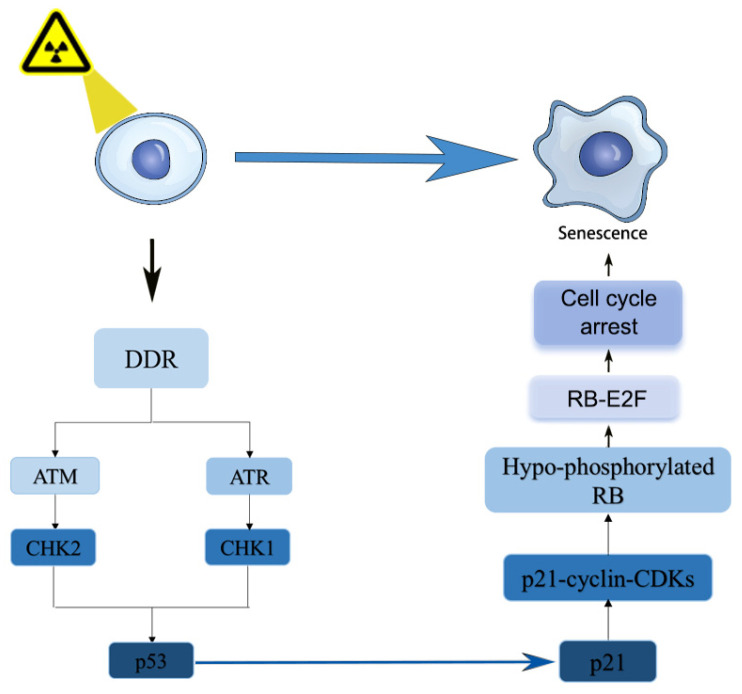
p53/p21 signaling (DNA damage → p53 ↑ → p21 ↑ → CDK inhibition → cell cycle arrest → senescence) in IR-induced BM-MSC senescence (created with Adobe Illustrator 28.5).

TP53 can be phosphorylated at multiple sites by ATM to inhibit its degradation [52], and phosphorylated p53 has been reported to regulate the cell cycle arrest, senescence and apoptosis in DDR [53]. Interestingly, negative regulators of p53 such as murine double minute 2 (MDM2) and MDM4 can also be phosphorylated by ATM [54]. Phosphorylated MDM2 and MDM4 become positive regulators of p53, inhibiting its ubiquitination and promoting the stabilization and activation of p53 [55]. Detailed descriptions of the DDR signaling pathways have been well summarized in previous studies [45,55,56]. The accumulation of p53 induces cell cycle arrest, DNA repair, senescence and/or apoptosis by transcriptional activation of downstream target genes [57]. The most important downstream gene of p53 is the cyclin-dependent kinase inhibitor 1A (CDKN1A) [58], also known as p21, which inhibits the activity of mitotic CDKs to modulate cell fate [59]. Through negative regulation of the CDK4- and CDK6-driven phosphorylation of retinoblastoma protein (RB), p21 prevents the release of E2F1, thereby repressing the transcription of E2F1-driven G1/S phase-promoting genes [60]. In astrocytes and osteoblasts, p21 is also involved in p53-independent DNA damage-induced G2 arrest, mediated by CREB3L1 [61]. Another CDK inhibitor, CDKN2A (also known as p16), can control the activity of RB by inhibiting CDK4/6, leading to cell cycle arrest in the G1 phase. The p53-p21 and p16-RB pathways are the most extensively studied mechanisms for cell cycle arrest-induced cellular senescence. The activation of these two pathways may depend on the cell type and the inducer [62]. In some cell types, p21 is activated early to initiate cell cycle arrest and declines over time, while p16 is activated later, as p21 declines, to maintain senescence [63]. This dynamic balance between p21 and p16 expression critically influences the timing and maintenance of the senescent state. The DDR cascade, mediated by PIKKs and checkpoint kinases, converges on p53 activation to regulate this process. Notably, in BM-MSCs, the p53-p21 pathway serves as a central mechanism for IR-induced senescence [64], integrating DNA damage signals with cell cycle arrest (Figure 2).

However, cell cycle arrest is not senescence [65]. Senescent phenotypes emerge gradually, only under prolonged cell cycle arrest driven by DNA damage, coupled with the continued activation of growth-promoting pathways such as the mammalian target of rapamycin (mTOR) and mitogen-activated extracellular signal-regulated kinase (MEK)/mitogen-activated protein kinase (MAPK), despite blocked division. These combined signals ultimately lead to altered cell morphology, SA-β-gal positivity, and SASP secretion [66]. Thus, persistent DNA damage caused by IR can induce cellular senescence [67]. Senescence arises when DNA damage-induced arrest is sustained and accompanied by constitutive growth signaling, leading to irreversible phenotypic changes.

### 2.2. Oxidative Lesion-Induced Cellular Senescence

Besides direct DNA damage, IR can also induce the immediate production of ROS in the cell by water radiolysis. These free radicals cause oxidative damage to nuclear DNA (nDNA), mitochondrial DNA (mtDNA), and proteins [68]. The oxidative damage to mtDNA and proteins further causes mitochondrial dysfunction, which can induce cellular senescence [69]. Therefore, mitochondrial dysfunction is both a hallmark and a driver of cellular senescence and aging [70].

In physiological conditions, ROS released from mitochondria are not a significant source of oxidative DNA damage [71]. However, upon exposure to IR, continuous generation of ROS (e.g., ${•O}_{2}^{-}$, H_2_O_2_, •OH and organic radicals) induces persistent oxidative nDNA damage in normal cells, which may persist for days to months, leading to permanent cell cycle arrest and cellular senescence [68]. Concurrently, mitochondrial dysfunction occurs, characterized by loss of mitochondrial membrane potential, increased mitochondrial mass, alterations in respiratory chain complexes, cytochrome c (Cyt c) release, reduced mitochondrial transcription factor A (TFAM) expression and mtDNA damage [72]. This dysfunction drives cellular senescence through the nicotinamide adenine dinucleotide (NAD)-AMP-activated protein kinase (AMPK)-p53 (NAD-AMPK-p53) pathway and enhances SASP secretion [73]. Notably, mitochondrial elimination through mitophagy suppresses SASP but fails to reverse the cell cycle arrest in senescent cells [74]. IR-induced oxidative stress damages the nuclear and mitochondrial components. Mitochondrial dysfunction amplifies ROS and activates NAD–AMPK–p53 signaling, promoting senescence and SASP.

Recent studies report that mtDNA damage may be the main reason for cellular senescence under oxidative stress [75,76]. Compared with nDNA, mtDNA is more vulnerable to oxidative damage, probably due to the lack of histone binding protection, deficiency of traditional repair way, and proximity to the electron transport chain [72]. When mitochondrial outer membrane permeabilization (MOMP) occurs in a small subset of mitochondria without cell death, termed minority MOMP [77], damaged mtDNA is released into the cytosol via the macropores formed by BCL-2-associated X protein (BAX) and BCL-2 antagonist/killer (BAK1) [78,79], triggering downstream inflammatory responses [76,80]. Recently, Passos et al. demonstrated that the mitochondrial permeability transition triggered by BAX leads to the leakage of TFAM-bound mtDNA nucleoids, which contributes to the secretion of SASP [76]. However, this process can be prevented by the inhibition of BAX. Additionally, another mechanism of mtDNA damage inducing senescence is the activation of the cyclic GMP-AMP synthase (cGAS) stimulator of the interferon genes (STING) (cGAS-STING) pathway, which is involved in DNA immune sensing and plays a significant role in cellular senescence [81]. Furthermore, Xu et al. reported that the cGAS-STING-PKR-like endoplasmic reticulum kinase (PERK)-eukaryotic initiation factor 2 alpha (eIF2α) (cGAS-STING-PERK-eIF2α) pathway initiates the cellular senescence [82], while the cGAS-STING-interferon regulatory factor 3 (IRF3)-RB (cGAS-STING-IRF3-RB) pathway reinforces senescence-related phenotypes [83]. mtDNA damage and cytosolic release activate immune-sensing pathways such as cGAS–STING, which drive inflammatory senescence phenotypes and SASP secretion.

Beyond its well documented effects on DNA and mitochondria, IR also damages other organelles such as the ER, lysosomes, and the Golgi apparatus [84]. However, the contributions of these alterations to radiation-induced cellular senescence are not as well understood [22,85,86]. Consequently, this review will focus exclusively on the mechanisms of DNA damage and mitochondrial dysfunction in driving senescence in BM-MSCs.

## 3. IR-Induced BM-MSCs Damage

BM-MSCs are resistant to IR-induced apoptosis [4]. When they are exposed to γ-radiation, the DDR pathways are rapidly and effectively activated to facilitate DNA repair and promote cell survival [5]. Upon IR, the γH2AX foci form rapidly in BM-MSCs but are also efficiently resolved within 24 h [87]. Moreover, key DDR proteins such as ATM, DNA-PK, and CHK2 are constitutively and highly expressed in BM-MSCs [88]. In mouse models, BM-MSCs exhibit an intrinsic anti-apoptotic phenotype, characterized by high expression of proteins like Bcl-2 and Bcl-xL and low expression of pro-apoptotic proteins such as Bim and Puma [88]. Similarly, human BM-MSCs exhibit elevated expression of anti-apoptotic proteins and a heightened antioxidant capacity, both of which contribute to their radioresistance [5,89].

BM-MSCs are also susceptible to radiation injury in a dose-dependent way [90]. Prior to allogeneic or autologous hematopoietic stem cell transplantation, total body irradiation (TBI) is commonly used as part of the conditioning regimen. Typically, a fractionated dose of 2 Gy per fraction is delivered twice daily over three consecutive days, totaling 12 Gy [91]. Although TBI does not significantly alter the morphology, surface marker expression, or hematopoiesis-supporting function of BM-MSCs in leukemia patients and healthy adults, it profoundly impairs their proliferative capacity and chromosomal integrity [92]. Furthermore, TBI simultaneously suppresses the adipogenic and osteogenic differentiation abilities of BM-MSCs, rather than selectively shifting the balance between these two lineages [92]. In co-culture experiments, the expansion of hematopoietic stem and progenitor cells (HSPCs) and the production of mature myeloid cells are not affected by irradiated BM-MSCs, which is consistent with the observation that the hematopoiesis-supporting function of BM-MSCs remains largely intact after TBI [93]. However, B-cell production is significantly suppressed in co-cultures with irradiated BM-MSCs. This impairment is attributed to the downregulation of CXCL12 and IL-7, which are both essential for B-cell lymphopoiesis, which persists for approximately 2 to 3 weeks after γ-irradiation [93]. Conversely, our group observed that the equilibrium between adipogenic and osteogenic differentiation of human BM-MSCs was significantly disrupted following a single 9 Gy γ-irradiation (Cobalt-60), with cells exhibiting a strong tendency toward adipogenesis via the CR6-interacting factor 1 (CRIF1)-protein kinase A (PKA)-cAMP response element binding protein (CREB) (CRIF1-PKA-CREB) pathway [94]. These results reveal the complex, dose-dependent effects of irradiation on BM-MSCs: high-dose irradiation (9 Gy) disrupts the adipogenic–osteogenic equilibrium and low-dose irradiation (2 Gy) more broadly impairs BM-MSCs function (including differentiation ability and the ability to support B-cell lymphopoiesis), while largely preserving their support for myeloid hematopoiesis.

In addition to the aforementioned cellular injuries, high-dose IR induces high levels of DNA damage and oxidative stress, which are key triggers for BM-MSCs senescence [7]. The work by Cmielova’s group provided a clear demonstration of the dose-dependent nature of this phenomenon: BM-MSCs recovered from 2 Gy γ-rays (Cobalt-60) within two weeks, whereas 20 Gy irradiation triggered permanent cell cycle arrest in the G2/M phase, indicating irreversible senescence [95]. Beyond the dose, the radiation type also critically determines the pattern of DNA damage [96]. High-LET radiation results in complex and clustered DNA lesions that are repaired more slowly and less efficiently than damage caused by low-LET radiation [97,98]. Therefore, the extent and underlying mechanisms of BM-MSC senescence are critically influenced by both the dose and the type of IR [99], resulting in a spectrum of responses (Table 1). In the forthcoming sections, the mechanisms of radiation-induced senescence in BM-MSCs will be discussed by classifying radiation according to its biological effectiveness and damage complexity: γ-ray and X-ray radiation (low-LET) and heavy-ion radiation (high-LET).

### 3.1. γ-Rays Radiation-Induced BM-MSCs Senescence

Exposure to low-dose (0.1 Gy) γ-radiation from Cesium-137 delays the cell cycle of human BM-MSCs. However, it is difficult to find the related morphology changes. In contrast, BM-MSCs exposed to 4 Gy γ-radiation display senescent morphologies [107]. Cmielova and colleagues showed that γ-radiation (Cobalt-60) at doses of 2 Gy and 6 Gy reduced the number of BM-MSCs in the S phase on the first day post-irradiation, and that doses of 6 Gy and 20 Gy led to substantial accumulation of BMC-MSCs in the G2/M phase [95]. They also reported that the proportion of BM-MSCs undergoing cell cycle arrest decreased by nearly half at 6 Gy six days after irradiation, but remained largely unchanged at 20 Gy. Within two hours after exposure to 9 Gy Cobalt-60 γ-rays, our group observed an accumulation of BM-MSCs in the G2 phase and an increased SA-β-gal activity [108]. We also found that the ROS level peaked within 4 h after irradiation; although it partially recovered within 24 h, it remained significantly elevated compared to the baseline levels [109]. Schönmeyr et al. investigated the effects of a single dose of either 7 Gy or 12 Gy γ-radiation (Cesium-137) on the proliferation, cell cycle progression, senescence, differentiation, and gene expression of rat BM-MSCs [100]. The extent of proliferation, the proportion of cells in G2 phase arrest, the duration of arrest and SA-β-gal activity were all dependent on the radiation dose. Fekete et al. observed that a large portion of BM-MSCs failed to recover after exposure to 30 Gy (sublethal) or 60 Gy (lethal) Cesium-137 γ-radiation, as evidenced by apoptotic bodies and loss of cell adhesion [102]. Notably, a small subpopulation of cells survived and retained proliferative ability for up to 120 days. The surviving cells exhibited permanent cell cycle arrest in the G1 phase and showed detectable SA-β-gal activity by day 10 following 30 Gy or 60 Gy radiation.

In rat models, exposure of BM-MSCs to γ-radiation (Cesium-137) at doses of 2 Gy, 5 Gy, and 10 Gy triggered distinct biological responses [101]. At 2 Gy, a slight reduction in cell viability and colony-forming ability was observed, while these effects were more significant at 5 Gy and 10 Gy. Irradiation led to DNA damage, an increase in SA-β-gal positive cells, upregulation of p21, and reduced expression of antioxidant enzymes. Furthermore, irradiation also activated the Janus kinase 1 (JAK1)-signal transducer and activator of transcription 3 (STAT3) (JAK1-STAT3) pathway, resulting in enhanced secretion of SASP components such as IL-6, IL-8, MMP-9 and MMP-12. The irradiated cells underwent morphological changes and displayed decreased viability and differentiation potential. These phenomena became more significant as the radiation dose increased.

### 3.2. X-Ray Radiation-Induced BM-MSCs Senescence

Individual SSBs and DSBs are two major types of DNA lesions generated by X-rays. Most of these lesions can be repaired within 24 h. The remaining damage, which is predominantly localized in telomeric DNA, may lead to cellular senescence [37].

X-ray-induced senescence in human BM-MSCs is also dose-dependent. For instance, after exposure to 4 Gy X-ray (320 kVp), approximately 85% of BM-MSCs exhibited enlargement, flattening, and positive SA-β-gal staining on day 3, compared to only 20% of the sham-irradiated cells (0 Gy) on day 10 [105]. This phenomenon may be attributed to proliferative senescence [110]. In terms of cell cycle arrest, both 1 Gy and 3 Gy X-ray irradiation induced G2/M arrest within 5 h. The G2/M arrest caused by 1 Gy radiation had largely recovered and progressed to the G1 phase after 24 h. However, BM-MSCs treated with 3 Gy radiation remained in persistent G2/M arrest [104]. At the molecular level, p53 was activated through Ser15 phosphorylation within minutes and was followed by upregulation of p21 expression after irradiation. These results suggest that the p53/p21 pathway plays a crucial role in the initial X-ray-induced cell cycle arrest. Additionally, 4 Gy X-ray radiation led to a significant upregulation of several cytokines and chemokines, such as growth-related oncogene (GRO), IL-8, IL-12, and macrophage-derived chemokine (MDC). These molecules are known as NF-κB responsive genes, which suggests activation of the NF-κB pathway during radiation-induced senescence [111].

### 3.3. Heavy-Ion Radiation-Induced BM-MSCs Senescence

In contrast to the predominantly isolated DNA lesions induced by low-LET photons, high-LET heavy-ion radiation is characterized by its ability to generate complex and clustered DNA damage, which fundamentally alters the ensuing cellular response in BM-MSCs. These complex lesions include clustered damage and simultaneous lesions at multiple sites, which are more difficult to repair efficiently [112]. Moreover, the biological effects of heavy-ion radiation on human BM-MSCs differ significantly from those induced by photon radiation [104,113], such as altered DDR, cell cycle arrest, differentiation capacity, and secretory profiles.

Almeida-Porada et al. reported distinct gene expression profiles in human BM-MSCs following irradiation with protons, iron-56 (^56^Fe) ions and γ-rays [113]. Notably, gene expression changes induced by 100 cGy of γ-radiation (Cesium-137) largely returned to pre-irradiation levels after five passages in culture. In contrast, alterations triggered by protons or ^56^Fe ion radiation persisted through multiple cell divisions, suggesting that heavy-ion radiation induces stable changes transmitted to clonal daughter cells. Furthermore, the expression changes in most cytokines, and chemokines were highly dependent on radiation type. Consequently, the senescence phenotype of BM-MSCs induced by heavy-ion radiation likely differs fundamentally from that induced by γ-rays, particularly in terms of persistence, mechanisms involving DDR, and functional consequences for the hematopoietic niche.

Recently, the Nicolay group studied the effects of various high-LET particle radiation on human BM-MSCs [14]. They found that radiation with 2 Gy protons, 1 Gy helium (^4^He), 0.8 Gy carbon (^12^C) and 0.8 Gy oxygen (^16^O) ions was biologically equivalent to 2 Gy of X-ray radiation. This particle radiation induced a significant increase in phosphorylation of ATM and CHK2 in human BM-MSCs at 2 h post-irradiation. Although residual phosphorylated ATM could still be detected after 24 h, its level had significantly decreased. Meanwhile, the mean number of γH2AX foci per nucleus increased significantly at 2 h and returned to near-baseline levels at 24 h post-irradiation. The authors also investigated the cell cycle arrest induced by particle radiation. At 96 h post-irradiation, only a slight G2/M cell accumulation was observed in two out of three BM-MSC samples. The expression of p21 was not significantly upregulated after irradiation, which may be due to the high baseline expression in untreated cells. Although some variation was observed among different BM-MSC specimens, apoptosis of BM-MSCs was low under all radiation types. All these results indicated that low-dose particle radiation did not induce significant alterations in BM-MSC functional properties, differentiation potential, or cellular morphology. This demonstrated the relative radioresistance of BM-MSCs to moderate doses of high-LET radiation.

Owing to the small number of cells hit by alpha particles at low doses, 40 mGy alpha particle radiation (from Americium-241, LET = 112 keV/μm) induced significantly fewer senescent cells and negligible apoptosis compared with X-rays at the same dose [106]. Following alpha particle irradiation, ATM immunostaining increased initially but declined to nearly the control by 48 h. At an intermediate dose level (2000 mGy), alpha particle irradiation increased the number of senescent cells, which was still fewer than that induced by X-rays at the same dose. Notably, due to the complex and clustered nature of DNA damage caused by high-LET alpha radiation, DNA repair was more challenging, leading to persistently higher levels of the DNA damage marker γH2AX, sustained ATM activation, and relatively higher apoptosis at 48 h post-irradiation compared with X-rays. In terms of cell cycle arrest, alpha particle radiation at doses of 40 and 2000 mGy reduced the proportion of S-phase cells and led to an accumulation of G0/G1 phase cells. While exposure to 40 mGy alpha particles slightly reduced the number of G2/M phase cells, 2000 mGy irradiation resulted in a marked increase in G2/M phase cells at 6 and 48 h after treatment.

The Wang group [104] investigated the effects of high-LET ^56^Fe ions (1 GeV/amu, LET = 150 keV/μm) and X-rays on human BM-MSCs. They reported that 1 Gy of ^56^Fe ion radiation significantly reduced the proportion of S phase cells, slightly decreased the G1/G0 phase fraction, and caused substantial G2/M phase accumulation. The G2/M arrest caused by 1 Gy ^56^Fe ion radiation was more severe than that induced by 3 Gy X-rays, consistent with the notion that DNA damage from high-LET radiation is more complex and challenging to repair. Bioinformatics analysis revealed that key cell-cycle-related genes, including CCNB1, CCNE2, and HELLS, were markedly downregulated following exposure to 0.1 Gy ^56^Fe ions, whereas these genes exhibited only a minimal response to an equivalent dose of X-rays. The expression of p53 and p21 was also more strongly induced by ^56^Fe ions than X-rays. Therefore, high-LET radiation proved to be a more potent inhibitor of mitosis in BM-MSCs compared with X-rays.

## 4. Senotherapeutic Strategies for BM-MSC Senescence

Therapies targeting senescent cells comprise four categories: senolytics, senomorphics, senoblockers and senoreversers [114]. Senolytics, such as Dasatinib plus Quercetin (D + Q) [115], and Fisetin [116], selectively eliminate senescent cells by targeting senescent cell anti-apoptotic pathways, including inhibition of the BCL-2 family [117]. Senomorphics, including Metformin [118], Rapamycin [119], and Spermidine [120], modulate the SASP by inhibiting pathways such as mTOR or NF-κB, promoting autophagy, and reducing chronic inflammation [121]. Senoblockers, such as Bruton’s tyrosine kinase (BTK) inhibitors (e.g., Ibrutinib), prevent the induction of cellular senescence by disrupting pathways such as p53 stabilization [122]. Senoreversers, such as the small molecule reversine (an Aurora B kinase inhibitor) [123] or partial reprogramming using OSKM factors (OCT4, SOX2, KLF4, c-MYC) [124], aim to reverse the senescent phenotype by restoring youthful gene expression patterns, metabolic function, and nucleocytoplasmic compartmentalization [125]. The above treatment strategies are applicable to replication-induced, stress-induced, and disease-induced senescence in BM-MSCs, as summarized in Table 2, Table 3, Table 4 and Table 5, respectively.

IR-induced senescence in BM-MSCs can also be intervened through these strategies. For example, following local abdominal irradiation of rats with 14 Gy X-rays (Siemens PRIMUS), administration of Ginsenoside Rg1 (Rg1) upregulates heme oxygenase-1 (HO-1), thereby enhancing the antioxidant and anti-apoptotic capacity of BM-MSCs and ameliorating radiation-induced intestinal injury through enhanced paracrine support in vivo [138]. Similarly, in a mouse model employing 5 Gy TBI with γ-rays (Cesium-137), continuous intraperitoneal injection of the dietary antioxidant, ferulic acid (50 mg/kg/day for 37 days), mitigates BM microenvironment disruption and bone loss by scavenging ROS, boosting endogenous antioxidant enzymes (superoxide dismutase (SOD), catalase (CAT), glutathione peroxidase (GPx)), and inhibiting senescence in both HSCs and MSCs [141]. In another study [140], mice subjected to a sublethal dose (7.5 Gy, Cesium-137) of TBI and treated with the water-soluble radiation mitigator MMS350 (provided continuously in drinking water) alleviated radiation-induced senescence by clearing ROS and directly reducing the number of senescent cells. In a separate experiment, local irradiation of the left hind limb with 12 Gy X-rays followed by intraperitoneal delivery of the STING inhibitor C-176 (750 nmol per mouse, ~30 mg/kg) decreased the SASP factor (IL-6, RANKL) secretion, suppressed osteoclast activation, and attenuated inflammatory bone damage via inhibition of the STING–TBK1 pathway [139]. In vitro irradiation of rat BM-MSCs with Cesium-137 γ-rays (2–10 Gy doses) induced cellular senescence. These senescent BMSCs released SASP factors (such as IL-6, IL-8, MMP-9) via paracrine signaling, which activated the JAK1/STAT3 pathway and subsequently inhibited osteogenic differentiation, culminating in impaired bone formation. Treatment with a JAK1 inhibitor effectively suppressed phosphorylation in the JAK/STAT3 pathway, reduced the secretion of SASP components, improved osteogenic dysfunction, and ultimately alleviated radiation-induced senescence in BM-MSCs [101].

Despite these promising findings, senotherapeutic applications in IR-induced BM repair face substantial challenges. The primary limitations include radiation-induced senescent cell heterogeneity and their disruption of hematopoietic signaling pathways, complicating simultaneous achievement of targeted elimination and functional recovery. Furthermore, treatment risks such as off-target toxicity, secondary inflammation, and sensitivity to timing windows impact safety profiles. Clinical translation is hindered by patient variability, combination therapy standardization difficulties, and reliable biomarker scarcity. Future efforts should focus on developing bone marrow-specific delivery systems, applying multi-omics technologies to identify radiation-specific senescence markers, and exploring rational combination strategies to achieve safe clinical application.

## 5. Conclusions and Perspectives

In summary, ionizing radiation (IR) induces senescence in BM-MSCs. This process is driven by the synergistic interplay between DNA damage and mitochondrial dysfunction. The severity and type of damage are critically modulated by the radiation parameters, including LET and dose. Comparative studies demonstrate that high-LET radiation (e.g., heavy ions) causes complex, clustered DNA lesions. These lesions challenge cellular repair pathways. In contrast, low-LET radiation (e.g., γ-rays, X-rays) primarily induces isolated lesions through ROS-mediated mechanisms. Mitochondrial dysfunction not only amplifies oxidative stress but is also exacerbated by it, creating a vicious cycle that collectively promotes senescence over apoptosis due to BM-MSCs’ inherent radio-resistance conferred by high anti-apoptotic protein expression. Various senotherapeutic strategies (e.g., senolytics, senomorphics, senoblockers) theoretically target this process. However, their efficacy is limited by radiation’s multidimensional damage. This damage simultaneously affects DNA integrity, epigenetic regulation, and proteostasis across cellular compartments. The experimental evidence from various models (summarized in Table 1, Table 2, Table 3, Table 4 and Table 5) underscores the necessity of developing radiation-type-specific interventions. Addressing how to repair such integrated damage remains the central challenge, highlighting the need for novel approaches that account for the distinct biological effects of different radiation qualities on BM-MSCs to effectively mitigate senescence-related pathologies.

## Figures and Tables

**Table 1:** The impact of various IR types and doses on BM-MSCs.

Source of BM-MSC	IR Source	IR Type	IR Dose	Cell Cycle Arrest	SASP Secretion	Proposed Mechanism of Senescence	Ref.
Rat	Cesium-137	γ-rays	7, 12 Gy	G2	/*	p53/p21	[100]
Human	Cobalt-60	γ-rays	2, 6, 20 Gy	G2/M	/	p53/p21, p16/RB	[95]
Rat	Cesium-137	γ-rays	2, 5, 10 Gy	G0/G1	IL-6, IL-8, MMP9, MP12	p53/p21, JAK1-STAT3	[101]
Human	Cesium-137	γ-rays	30, 60 Gy	G1	IL6	p53/p21, p16/RB	[102]
Human	6 MeV	X-rays	40, 160, 640, 2000 mGy	G2/M by 6 h; G0/G1 by 48 h	/	p53/p21	[103]
Human	320 kVp	X-rays	0.1, 1, 4 Gy	G2/M	IL-8, IL-12	p53/p21	[104,105]
Human	112 keV/μm, Americium-241	alpha particle	40, 2000 mGy	G0/G1, G2/M	/	/	[106]
Human	XRAD 320, 6 keV/μm ^1^H, 17 keV/μm ^4^He, 101 keV/μm ^12^C, 154 keV/μm ^16^O	X-rays, ^1^H, ^4^He, ^12^C, ^16^O	2Gy, 2 Gy ^1^H, 1 Gy ^4^He, 0.8 Gy ^12^C, 0.8 Gy ^16^O	G2/M	/	/	[14]
Human	1 GeV/amu, 150 keV/μm	^56^Fe	0.1, 1 Gy	G2/M	/	p53/p21	[104]

* ‘/’ represents Not mentiond.

**Table 2:** Senotherapeutic strategies for replication-induced senescence in human BM-MSCs.

Source of BM-MSCs	Trigger of Senescence	Senotherapeutic Strategies	Senotherapeutic Mechanisms	Ref.
Human	Replication in vitro	Quercetin treatment	Inhibition of Wnt/β-catenin signaling, upregulation of nuclear γ-catenin.	[126]
Human	Replication in vitro	ABT-263 (Navitoclax) treatment	Senolytic effect by inducing apoptosis in senescent cells.	[127]
Human	Replication in vitro	Melatonin	Preservation of stemness, inhibiting p16, p21, upregulating NANOG expression.	[128]
Human	Replication or metabolic dysfunction	Proline	Activates AMPKα-Parkin pathway to enhance mitophagy, restores mitochondrial respiration, and downregulates p53, p21.	[129]
Human	Replication in vitro	Young small extracellular vesicles from remnant pulp of human exfoliated deciduous teeth stem cells	Promotion of Drp1 translocation to mitochondria and restoration of mitochondrial fission.	[130]

**Table 3:** Senotherapeutic strategies for replication-induced senescence in animal BM-MSCs.

Source of BM-MSCs	Trigger of Senescence	Senotherapeutic Strategies	Senotherapeutic Mechanisms	Ref.
Rat	Replication in vitro	3-butyl-1-chloro imidazo [1,5-a] pyridine-7-carboxylic acid treatment	Promotion of lysosomal acidification, upregulation of LAMP1/LAMP2, inhibition of p21, reduction in SA-β-gal, promotes autophagy.	[131]
Rat	Replication in vitro	Aspirin treatment	Modulation of immune response and lipid metabolism.	[132]
Rat	Replication in vitro or H_2_O_2_ stimulation	Cholesterol treatment	Regulation of autophagy and ROS/p53/p21 pathway.	[133]
Canine	Replication in vitro	Curcumin treatment	Promotion of autophagy and lysosomal acidification, downregulation of p16, p21 and SASP.	[134]
Rat	Replication in vitro	Melatonin	Preservation of stemness, inhibiting p16, p21, upregulating NANOG expression.	[128]
Rat	Replication in vitro	Umbilical cord MSC-derived exosomes	Activation of autophagy via PI3K/AKT/mTOR pathway inhibition.	[135]
Mouse	Nature aging	Senolytic cocktail (Dasatinib + Quercetin)	Elimination of senescent cells.	[136]
Mouse	Nature aging	Local delivery of tetramethylpyrazine	Modulation of Ezh2-H3k27me3 and inhibition of p16.	[137]

**Table 4:** Senotherapeutic strategies for stress-induced senescence in BM-MSCs.

Source of BM-MSCs	Trigger of Senescence	Senotherapeutic Strategies	Senotherapeutic Mechanisms	Ref.
Rat	X-rays	Ginsenoside Rg1-preconditioned BMSC-conditioned medium	HO-1 upregulation; enhanced secretion of VEGF/IL-6, inhibition of NF-κB and apoptosis	[138]
Mouse	X-rays	STING inhibitor (C-176)	Downregulates STING-TBK1 pathway, inhibits SASP, reduces osteoclastogenesis, reduces DNA damage-induced senescence	[139]
Rat	γ-rays	JAK1 inhibitor (JAKi) treatment	Inhibition of JAK1/STAT3 pathway and SASP secretion	[101]
Mouse	TBI, replication in vitro or Fanconi anemia genotype	Radiation mitigator MMS350	Antioxidant and radiation protector	[140]
Mouse and rat	TBI	Ferulic acid	Enhanced endogenous antioxidant defense (SOD, CAT, GPx), reduced ROS, NRF2 upregulation	[141]
Mouse and human	1,25-dihydroxyvitamin D deficiency or aging	1, 25(OH)_2_D_3_	Upregulation of Ezh2-H3k27me3 and inhibition of p16/p19	[142]
Mouse	Doxorubicin-induced and aging	Bone-targeted delivery of quercetin	Elimination of senescent cells	[143]
Human	H_2_O_2_, aging, or mTOR hyperactivation	Recombinant Indian Hedgehog protein	Inhibition of ROS/mTOR/4EBP1/p70S6K pathway, reduced p53/p16	[144]
Rat	H_2_O_2_-induced oxidative stress	Quercetin treatment	Inhibition of repetitive element (RE) activation and retinoic acid-inducible gene I (RIG-I) RNA sensing pathway	[145]
Human and rat	H_2_O_2_-induced oxidative stress	Erxian Decoction (EXD)-derived exosomes	Activation of mitophagy (PINK1/Parkin pathway), reduced ROS, enhanced mitochondrial membrane potential	[146]
Rat	TNF-α-induced oxidative stress and mitochondrial damage	Salidroside	Induces Parkin-mediated mitophagy via AMPK activation, clears damaged mitochondria, and reduces ROS/DNA damage	[147]
Mouse and rat	Iron overload, inducing oxidative stress and mitochondrial dysfunction	Melatonin	Reduction in ROS, prevention of mitochondrial membrane potential depolarization, inhibition of p53/ERK/p38 pathways	[148]
Human	NAD^+^/NADH imbalance and mitochondrial dysfunction	Nicotinamide	Rebalances NAD^+^/NADH ratio, activates SIRT1, enhances mitophagy, and restores mitochondrial fitness	[149]
Rat	Natural aging or D-galactose exposure	Zuogui Wan (ZGW) herbal formula	Downregulates Wnt/β-catenin signaling, inhibits p16/p21 and SASP, promotes mitochondrial biogenesis	[150]
Human	D-galactose (D-gal)-induced senescence	Metformin treatment	Activation of AMPK pathway; enhancement of autophagy flux to reduce ROS, restore mitochondrial membrane potential, and reverse cell cycle arrest	[151]

**Table 5:** Senotherapeutic strategies for disease-induced senescence in BM-MSCs.

Source of BM-MSCs	Trigger of Senescence	Senotherapeutic Strategies	Senotherapeutic Mechanisms	Ref.
Mouse	Chronic kidney disease (CKD), oxidative stress or DNA damage	Metformin	AMPK pathway activation, inhibition of NF-κB and SASP, decrease in DNA damage markers, downregulation of prelamin A	[152]
Rat	Estrogen deficiency (ovariectomized), oxidative stress or DNA damage	Eldecalcitol (ED-71) oral administration	Activation of SIRT1-Nrf2 signaling pathway, reduction in ROS, inhibition of p16/p53 expression	[153]
Rat	Estrogen deficiency, mitochondrial dysfunction or ROS accumulation	Genistein oral administration	ERRα-mediated mitochondrial biogenesis and mitophagy, upregulation of PGC1α/SIRT3	[154]
Rat	Estrogen deficiency and oxidative stress	Liuwei Dihuang pills	Activation of YAP-autophagy axis, upregulation of autophagy and YAP, downregulation of p62	[155]
Rat	Estrogen deficiency and oxidative stress	Melatonin	Activation of AMPK-SIRT1 pathway, reduction in p16, p21, p53	[156]
Rat	Aplastic anemia microenvironment, oxidative stress or MAPK activation	Icariin treatment	Suppression of MAPK pathway (p38/JNK/ERK), downregulation of PPARγ/C/EBPα/FABP4	[157]

## Data Availability

No new data were created or analyzed in this study. Data sharing is not applicable to this article.

## Abbreviations

BM-MSCs: Bone marrow mesenchymal stem/stromal cells; IR: ionizing radiation; ARS: acute radiation syndrome; BM: bone marrow; HSCs: hematopoietic stem cells; CXCL12: C-X-C motif chemokine 12; SCF: stem cell factor; PGE2: prostaglandin E2; IDO: indoleamine 2,3-dioxygenase; TGF-β: transforming growth factor-β; IL: interleukin; NK: natural killer; GVHD: graft-versus-host disease; ROS: reactive oxygen species; RNS: reactive nitrogen species; ER: endoplasmic reticulum; LET: linear energy transfer; DDR: DNA damage response; SA-β-gal: senescence-associated β-galactosidase; SASP: senescence-associated secretory phenotype; AML: acute myeloid leukemia; CLL: chronic lymphocytic leukemia; MM: multiple myeloma; SSBs: single-strand breaks; DSBs: double-strand breaks; BER: base-excision repair; SSBR: single-strand break repair; NHEJ: non-homologous end joining; HR: homologous recombination (HR); TMEJ: theta-mediated end joining; PIKKs: phosphatidylinositol-3 kinase-related kinases; ATR: ataxia-telangiectasia and RAD3-related; ATM: ataxia-telangiectasia mutated; DNA-PK: DNA-dependent protein kinase; CHK: checkpoint kinases; CDC25A: cell division cycle 25 homolog A; CDK: cyclin-dependent kinase; TP53, p53: tumor suppressor protein; γH2AX: phosphorylated forms of the histone variant H2AX; 53BP1: p53-binding protein 1; MDM2: murine double minute 2; CDKN1A, p21: cyclin-dependent kinase inhibitor 1A; RB: retinoblastoma protein; CDKN2A, p16: cyclin-dependent kinase inhibitor 2A; mTOR: mammalian target of rapamycin; MEK: mitogen-activated extracellular signal-regulated kinase; MAPK: mitogen-activated protein kinase; nDNA: nuclear DNA; mtDNA: mitochondrial DNA; Cyt c: cytochrome c; TFAM: mitochondrial transcription factor A; NAD: nicotinamide adenine dinucleotide; AMPK: AMP-activated protein kinase; MOMP: mitochondrial outer membrane permeabilization; BAX: BCL-2-associated X protein; BAK1: BCL-2 antagonist/killer; cGAS: cyclic GMP-AMP synthase; STING: stimulator of interferon genes; PERK: PKR-like endoplasmic reticulum kinase; eIF2α: eukaryotic initiation factor 2 alpha; IRF3: interferon regulatory factor 3; TBI: total body irradiation; HSPCs: hematopoietic stem and progenitor cells; CRIF1: CR6-interacting factor 1; PKA: protein kinase A; CREB: cAMP response element binding protein; JAK1: Janus kinase 1; STAT3: signal transducer and activator of transcription 3; MMPs: matrix metalloproteinases; GRO: growth-related oncogene; MDC: macrophage-derived chemokine; BTK: Bruton’s tyrosine kinase; OCT3/4: octamer-binding protein 3/4; KLF4: Krüppel-like factor 4; OSKM: OCT4, SOX2, KLF4, c-MYC; Rg1: Ginsenoside Rg1; HO-1: heme oxygenase-1; SOD: superoxide dismutase; CAT: catalase; and GPx: glutathione peroxidase.
